# Nutritional intake of sport undergraduates in Sabaragamuwa University of Sri Lanka

**DOI:** 10.1186/s40795-022-00662-0

**Published:** 2023-01-02

**Authors:** W. A. W. S. Rupasinghe, T. S. H. Perera, K. D. R. R. Silva, S. Samita, M. Nirmali Wickramaratne

**Affiliations:** 1 Department of Sports Sciences and Physical Education, Faculty of Applied Sciences, Sabaragamuwa University of Sri Lanka, Belihuloya, Sri Lanka; 2 Department of Applied Nutrition, Faculty of Livestock, Fisheries and Nutrition, Wayamba University of Sri Lanka, Makandura, Gonawila Sri Lanka; 3 Department of Crop Science, Faculty of Agriculture, University of Peradeniya, Peradeniya, Sri Lanka; 4 Department of Biochemistry, Faculty of Medicine, Sabaragamuwa University of Sri Lanka, Hidellana, Rathnapura Sri Lanka

**Keywords:** Macronutrient intake, Micronutrient intake, Energy intake, Dietary recommendations

## Abstract

**Background:**

Nutritional intake plays an important role in determining energy availability which is vital to health, wellbeing, and sports performance in an active population. This research assessed the sports undergraduates' nutritional intake compared to the Dietary Guidelines for Americans and nutrition goals provided by WHO.

**Methods:**

This study is a quantitative, cross-sectional descriptive study. One hundred and one (*n* = 101) sports undergraduates aged between 20 to 23 years were recruited and the nutrient intake was assessed using the three-day food diary method and quantified the macro and micronutrients by the food composition database. One sample t-test was performed to compare the mean nutrient intakes with the lowest recommendation values.

**Results:**

Though most undergraduates were able to meet the dietary requirements in carbohydrates, they were deficient in their protein intake and exceeded in fats intake. Further, both male and female students were deficient in their daily energy intake (1723 kcal, 1607 kcal) and dietary fiber intake (8 g, 11 g). The saturated fat intake was met by all students while 20% of males and 21% of females exceeded the recommendations (< 10%). The micronutrient intake of vitamins such as C, B1, B2, B9, and B12 and minerals such as Calcium, Magnesium, and Potassium, were significantly below the recommendations (*p* < 0.05) except for vitamin B3 niacin.

**Conclusions:**

Providing a nutritionally valuable meal is essentially required to maintain both physical and mental fitness. Our results revealed that the Sri Lankan sport science undergraduates do not have an adequate daily dietary intake of energy, proteins, calcium, magnesium, potassium, and vitamins such as C, B1, B2, B9, and B12.

## Background

A healthy diet, regular physical activities with optimum body composition, and physical fitness will foster lifelong health and wellness [1]. Further, the dietary requirements of athletes of various sports are significantly different from that of the Recommended Dietary Allowance (RDA) of a general population as their energy requirements vary [2, 3]. Therefore, students participating in intense physical activities and sports require special nutritional considerations that are sufficient in providing sufficient energy intake, and macro & micro-nutrients to meet the energy demands, and maintain physical and mental fitness, thereby optimizing both physical and academic performance [3]. Macronutrients provide the energy requirement and the micronutrients are essential in maintaining the proper metabolism; hence they are essential not only in the production of energy but also in tissue repair and recovery from injuries, transport of oxygen, maintaining bone strength, and reducing oxidative stress [4]. Further, nutrient deficiencies can lead to deficiency diseases and impair mental and physical health, and also increase susceptibility to injuries and illness, and decrease performance [5, 6].

A dietary guideline designed for a specific nation is aimed to improve the health and well-being of the population by incorporating lifestyle, eating patterns, and commonly available food to optimize health status and reduce nutritional deficiencies [7]. Several studies have been conducted for different populations (by ethnicity, by country, by profession) for the assessment of the nutritional status to obtain precise information the dietary intake and to develop nutritional guidelines and policies for the improvement of public health. The Nutritional division, of the Ministry of Health in Sri Lanka, has recognized the importance of developing such dietary guidelines for the country and are in the process of developing such guideline for the country’s general population. Further, the nutritional intake for carbohydrate, protein, and lipid levels have been identified by the Ministry of Health in Sri Lanka at 55—65%, 15–20%, and 30%, respectively [8]. However, to the best of our knowledge, there is no complete guideline for Sri Lankan population and the South Asian population. According to Dietary Guidelines for Americans (DGA), the average healthy individual's Acceptable Macronutrient Distribution Range (AMDR) of carbohydrates, lipids, and proteins are 45–65%, 20–35%, and 10–35%, respectively [7]. Several studies performed on nutritional analysis of athletes involved in different sports activities and compared with the dietary recommendations provided by organizations such as the International Society of Sports Nutrition (ISSN), American College of Sports Medicine [8], and the International Olympic Committee (IOC) [9], revealed that majority of athletes do not meet with the RDA of the macronutrients [10].

University undergraduates with heavy coursework and practical schedule mainly depend on the university cafeteria with a limited choice of food for their meals. Further, their dietary practices, habits, quantity, or quality are not monitored or guided; hence the food intake, in general, is less healthy with rich in carbohydrates and fats [9]. Previous investigations also revealed that most college students are unable to meet the Dietary Guidelines [10, 11]. Persistence of low energy intake would lead to a negative energy balance, impair physical performance, and lead to adverse health effects including chronic fatigue, electrolyte imbalance, weight loss, menstrual dysfunction, disruption of endocrine function etc. [12–14]. Carbohydrates, proteins, and fats are Macronutrients essentially required for growth, reproduction, maintaining immunity, healing, and regular maintenance of good health at large. Micronutrients that include vitamins and minerals are essentially required for energy productions to maintain immunity function, in growth, bone health, functions of the nervous system, etc. can be consumed through fruits and vegetables. Vitamin C promotes wound healing by facilitating collagen synthesis[15] and Vitamin D facilitate absorption of Calcium and bone growth. Vitamin D and Calcium are nutrients associated in maintaining muscle movements, nerve conduction, and healthy bones. Iodine along with other nutrients such as selenium and tyrosine are essentially required for the production of thyroid hormones thyroxine (T4) and triiodothyronine (T3) in the thyroid gland. Thyroid hormones are responsible for regulating body temperature metabolism and, therefore important in the mechanism of generating energy in athletes.

However, studies on the nutritional requirements of undergraduates who are supposed to be physically active as a compulsory requirement of the study discipline have not yet been adequately researched [16, 17]. Therefore, it is important to evaluate the sports undergraduates' nutritional parameters, who are expected to serve in the country's sports and physical education field.

The nutritional status can be assessed by dietary assessments with the estimation of nutrient intake (food recall procedures, dietary histories, and food records), anthropometric assessments by estimating physical dimensions, and body composition, clinical assessments evaluating medical history and physical examination of symptoms, biochemical assessments for the estimation of nutrient store metabolic functions, and excretions. Such nutritional assessment is important to identify critical nutrients required to develop a public health nutritional guideline to conduct nutritional awareness programs for the prevention of diseases related to nutrition [18]. The BMI measurement is a simple, less expensive, and non-invasive indicator of nutritional status, it is commonly used to obtain information on the energy input as well as it provides information on the obesity of an individual. BMI is correlated with body fat, fat-free mass, fat reserve, and proteins; hence it can be used to assess chronic energy deficiencies among adults, mostly in developing countries [19, 20]. The BMI is reported according to body weight, and height but does not provide much conclusive information. However, factors such as cormic index, age, and health issues like edema can influence BMI [21]. Since obesity is a leading cause resulting in diabetes, and circulatory and kidney diseases [15, 22, 23]has posed a burden in developing countries, especially among the youth [24]

Although several dietary recommendations are available for the general population prescribed by several institutions such as the United States Department of Health and Human Services & US Department of Agriculture [25], and WHO & FAO [26]; no such recommendations are available for the South Asian population or for Sri Lanka. Therefore, the present study was performed to assess the dietary intake of the sports undergraduates assessing the essential macro- and micro-nutrients over a three-day period, according to the recommended guidelines available by the WHO [26], US Department of Health and Human Services & US Department of Agriculture, and American College of Sport Medicine [25]. Further, to the best of our knowledge, this research would be the first study on the nutritional intake of sport undergraduates performed in Sri Lanka.

## Methods

### Subjects and study design

This study used a quantitative, cross-sectional descriptive research design. The department of Sport Sciences and Physical Education at the Sabaragamuwa University of Sri Lanka represents 67.1% of Sri Lankan sport undergraduates. All undergraduates (*n* = 255) who are following the BSc (Hons) Sport Sciences & Management or BSc (Hons) Physical Education at the Sabaragamuwa University of Sri Lanka in their first, second and third academic years in 2019 were invited for the study. Only 188 undergraduates volunteered to participate in the study, of which 164 undergraduates were eligible, qualifying for the eligibility screening. Those who reported any genetic disorder, on medication for metabolic disorders, cardiovascular diseases, cancers, diabetes, liver disorders, gastritis, on short-term medication during the test period and those who were pregnant or lactating were excluded from the study. Among 188 volunteer undergraduates, 164 undergraduates were eligible. However, only one hundred one (*n* = 101, 37male, 64 female) were included in the study after excluding the incomplete food dairies and who were detected as outliers.

### Ethical consideration

The ethical clearance was obtained from the committee on Ethics Review, Faculty of Allied Health Sciences, University of Peradeniya (AHS/ERC/2018/002). Further, informed consent from all participants was obtained before the commencement of the study.

### Data collection

#### Anthropometry

Body mass (kg) was measured using the Bioelectrical impedance Analyzer (Tanita-780, Japan), and standing height was measured using a stadiometer (Seca, 213, Germany).

#### Nutritional intake

The three-day food diary method was used to calculate the mean energy intake per day. It recorded all foods and beverages consumed on three consecutive days, including two weekdays and one weekend day, indicating the food type, preparation methods, quantities consumed, and brands. Further, smoking habits and dietary supplements used were also recorded. All volunteer participants were instructed about the dairy's completion and informed to keep the dairy with them all day. Instructions to complete the dairy were also printed in Sinhala, Tamil, and English languages to maintain the accuracy of their consumption. The recording freedom was ensured by allowing participants to record their dietary intake without any investigators being involved. The dairies that were incomplete were excluded from the study.

The dietary intake information received was analyzed through an electronic food composition database (FoodBase 2000; Institute of Brain Chemistry, University of London), the incomparable database on Sri Lankan foods. The macro and micronutrients were compared with the recommended dietary allowance (RDA), Acceptable Macronutrient Distribution Range (AMDR), or Adequate Intake (AI) with the Dietary Guidelines for Americans (DGA) provided by the US Department of Health and Human Services and U.S. Department of Agriculture [25], and Population nutrient intake goals provided by WHO [26].

### Statistical analysis

Statistical analysis was performed using the Minitab 18 software. The data were first checked for outliers using the Grubbs test and analyzed for normality using the Anderson–Darling test. Four respondents were removed from the analysis as outliers. For all variables, the normality test statics ranged between AD = 0.654 to 0.199 (*p* = 0. 081 to 0.880). Since the data satisfied the normality, one sample t-test was performed to determine the statistical significance between the minimum recommendation and the studied population mean in Macro and Micronutrients. *P* < 0.05 was considered statistically significant.

## Results

According to their BMI values, the majority of the male (54%) and female (55%) undergraduates' nutritional status was at their normal weight while 32% of males and 41% of females reported being in the underweight category. However, 11% of male and 6% of female students were overweight and 2% (one student) of female students were obese (Table 1).

**Table 1:** Nutritional Status by BMI ranges of the Sport Sciences Undergraduates

Nutritional Status	BMI Range^a^	Male n	Female n
Underweight	Below 18.5	13	24
Normal weight	18.5 – 24.9	20	35
Overweight	25.0 – 29.9	04	04
Obesity	Above 30.0	_-	01

*Source: *^*a*^ [23]

The mean nutrient intake of the male and female undergraduates was compared with the recommendations provided by the DGA and WHO for a general adult population (Table 2). The mean energy intake per day of male undergraduates was 1723 ± 296 kcal, whereas that of females was 1607 ± 365 kcal. Only 11% of males and 3% of females met with the minimum recommended energy intake/day compared to the recommended energy intake for males and females 2400 kcal and 2000 kcal respectively as provided by DGA (Table 2). On average, male undergraduates showed a significant deficit of energy by 677 kcal/day (t = 13.31, *p* = 0.000), while female undergraduates were significantly short of 393 kcal/day (t = -8.61, *p* = 0.000).

**Table 2:** Comparisons of nutrient intake with Dietary Guidelines for Americans and Nutritional goals by WHO

Nutrient Intake	Recommendation		Mean ± SD intake	
	**Female**	**Male**	**Female**	**Male**
			**(*n* = 64)**	**(*n* = 37)**
**Dietary Guidelines for Americans**^**a**^				
Energy, kcal/day	2000	2400	1607±365	1723±296
Protein, g (RDA)	46	56	44±10	49±10
Protein, % E (AMDR)	10–35	10–35	11±1	11±1
Carbohydrate, g (RDA)	130	130	252±61	273±49
Carbohydrate, % E (AMDR)	45–65	45–65	59±4	59±3
Dietary Fiber, g (AI)	28	33.6	10±7	9±3
Added Sugars, % E (DGR)	< 10	< 10	6±3	4±2
Total Fat, % E (AMDR)	20–35	20–35	30±4	29±3
Saturated fat, % E (DGR)	< 10	< 10	21±3	20±3
**Nutritional Goals provided by WHO**^**b**^				
Protein, % E	10–15		11±1	11±1
Carbohydrate, % E	55–75		59±4	59±3
Dietary Fiber, g	> 25		10±7	9±3
Added Sugars, % E	< 10		6±3	4±2
Total Fat, % E	15–30		30±4	29±3
Saturated fat, % E	< 10		21±3	20±3

*WHO* World Health Organization, *RDA* Recommended Dietary Allowance, *AMDR* Acceptable Macronutrient Distribution Range, *DGR* Dietary guidelines recommended limit, *AI* Adequate intake, *% E* Contribution for the total energy intake

^*^Considered 1800 kcal/day as the lower boundary of energy requirement, *Bw* Bodyweight, *n* = number of undergraduates

*Source: *^*a*^[25]*, *^*b*^[23]

### Energy intake and macronutrient intake

In comparison to the DGA’s RDA for the carbohydrate intake (130 g), both females (252 g, t = 15.97, *p* = 0.000) and males (273 g, t = 17.65, *p* = 0.000) showed a significantly higher intake being 193% and 210% high respectively. Further, according to the DGA’s AMDR (45–65%) and WHO nutritional goal (55–75%) % energy (E) requirement received from carbohydrates by all males (100%) and females (100%) were within the recommended ranges (Table 2).

However, the mean protein intake of female (44 g, t = -1.97, *p* = 0.027)) and male (49 g, t = -4.25, *p* = 0.000) undergraduates were significantly below the DGA recommendations as RDA for protein 46 g, and 56 g respectively. Therefore, only 19% of male and 39% of female students met with the DGA’s RDA of proteins. The mean % E requirement received from protein for both males and females was 11 ± 1 which is closer to the lower end of the recommendations (10–35). However, when comparing with the WHO recommendations of 10 – 15, percent energy (% E) from proteins, all (100%) male and female students were within the recommended range. Furthermore, according to the DGA recommendations as AMDR for protein (10–35%), 39% of females and 17% of males met the nutritional goal for protein. According to the nutritional goals for proteins provided by WHO (10–15%) 81% of females and 93% of males met the energy contribution through protein (Table 2).

The contribution of energy from total fat was met by 100% of both males and females according to recommendations of DGA (20–35%) and WHO (15–30%). However, according to WHO recommendations % E received from total Fat, 45% of females and 61% of males met the nutritional goals, while 55% of females exceeded by 2.9 ± 2.3% and 39% of males exceeded the total fat intake by 2.2 ± 1.9%. A 20% of males (t = 22.17, *p* = 0.000) and 21% of females (t = 26.66, *p* = 0.000) exhibited to be significantly higher mean intake of saturated fat than the recommendation of < 10% per day and females in comparison to both DGA’s DGR and WHO (Table 2).

All females and males did not consume an Adequate Intake (AI) of dietary fiber according to DGA's recommended 28 g and 33.6 g respectively. However, according to the WHO nutritional goals, only 8% of females met the recommendations of > 25 g per day. Therefore, the mean dietary fiber intake of males (9 g, t = -36.62, *p* = 0.000) and females (10 g, t = -16.08, *p* = 0.000) was significantly lower than the recommended intake of > 25 g/day (Table 2).

### Comparison of micro-nutrient intake with the recommendation

The micronutrients in the dietary intake of the undergraduates were compared with the recommendations provided by the WHO for both males and females in a general population (Table 3).

**Table 3:** Comparison of micronutrient intake with the WHO recommendation

Nutrient Intake	RDA		UL	(Mean ± SD) intake	
	**F**	**M**		**F** **(*n* = 64)**	**M** **(*n* = 37)**
Calcium, mg	1000^a^	1000^a^	2500	408±133	430±120
Magnesium, mg	220^a^	260^a^	350	180±44	188±40
Potassium, mg	> 3510^b^	> 3510^b^	-	1501±349	1538±301
Sodium, mg	< 2000^c^	< 2000^c^	2000	2424±603	2489±529
Iron, mg	19.6^a^	9.1^a^	45	9±2	10±2
Zinc, mg	4.9^a^	7.0^a^	40	6±1	7±1
Selenium, μg	26^a^	34^a^	400	44±16	55±19
Iodine ug	150^a^	150^a^	1100	56 ± 22	64 ± 22
Vitamin A ug	500^a^	600	3000	339 ± 265	353 ± 144
Thiamin, mg	1.1^a^	1.2^a^	-	0.5±0.1	0.6±0.2
Riboflavin, mg	1.1^a^	1.6^a^	-	0.5±0.2	0.5±0.2
Niacin, mg	14^a^	16^a^	35	16±4	18±4
Folate, μg	400^a^	400^a^	1000	180±56	198±55
Vitamin B6 mg	1.3^a^	1.3^a^	-	0.8 ± 0.2	0.9 ± 0.2
Vitamin B12, μg	2.4^a^	2.4^a^	-	1.4±0.5	1.3±0.4
Vitamin C, mg	45^a^	45^a^	2000	21±14	18±9
Vitamin D ug	5^a^	5^a^	100	0.9 ± 0.8	1.1 ± 0.8
Vitamin E mg	7.5^a^	10^a^	-	1.9 ± 1.7	1.8 ± 0.7

*RDA* Recommended Dietary Allowance, *AI* Adequate Intake, *UL* Upper Limit, *R* Recommendation, *M* Male, *F* Female

*Source: *^*a*^[27]*, *^*b*^*Guideline:* [28]*, *^*c*^*Guideline* [28]

### Mineral intake

The intake of Ca, and K was significantly low in all male and female students. Therefore, the Ca intake of females (408 mg, t = -35.6, *p* = 0.000), and males (430 mg, t = -4.25, *p* = 0.000) was significantly below the RDA of 1000 mg. Similarly, the K intake of females (1501 mg, t = -45.8, *p* = 0.000) and males (1538 mg, t = -39.7, *p* = 0.000) was significantly below the RDA of 3500 mg for both females and males. The Mg intake of females (180 mg, t = -7.3, *p* = 0.000) and males (188 mg, t = -10.9, *p* = 0.000) was significantly below the RDA of 220 mg for females and that of 260 mg for males. However, 16% of females and 02% of males meet the RDA for Mg. Further, none of the female students had the RDA for Iron (19.6 mg), while 66% of males met the RDA of 9.1 mg. The mean iron intake of males (10 mg, t = 2.74, *p* = 0.010) was significantly higher than the minimum RDA, while females' intake of 9 mg (t = -42.4, *p* = 0.000) was significantly lower than RDA. The sodium intake of both males (2489 mg) and females (2424 mg) was significantly higher (t = 5.6, *p* = 0.000) than the recommended upper limit of 2000 mg. Further, only 20% of females and 15% of males had their sodium intake within the recommended limit. No male or female undergraduates met the RDA for iodine dietary intake of 150 μg. The mean iodine intake of males (64 μg) and females (56 μg) were 43% and 37% lower than the recommendation, respectively. However, the recommended dietary intake was met for selenium by 93% of males and 91% of females. The mean Zn intake of females (6 mg, t = 8.8, *p* = 0.000), was significantly higher than the RDA of 4.9 mg, while males met with the RDA 7.0 mg. Further, 84% of females and 44% of males meeting with the daily Zn requirement (Table 3).

### Vitamin intake

The mean Vitamin A intake of females (339 μg) and males (353 μg) was significantly lower than the RDA of females 500 μg (t = -4.86, *p* = 0.000) and males 600 μg (t = -10.43, *p* = 0.000), respectively. Further, only 9% of females and 5% of males met the RDA for Vitamin A. The mean Vitamin E intake of females (1.9 mg) and males (1.8 mg) were 82% and 75% below the RDA of 7.5 mg and 10 mg, respectively. Further, the Vitamin E intake of females (1.9 mg, t = -26.35, *p* = 0.000) and males (1.8 mg, t = -71.26, *p* = 0.000) were significantly below the recommendation while none of the females or males met the daily Vitamin E requirement. The mean Vitamin D intake of females (0.9 μg, t = -41.00, *p* = 0.000) and males (1.1 μg, t = -29.65, *p* = 0.000) was significantly below the RDA of 5 μg and none of the females and males were met the RDA of Vitamin D intake. The Vitamin C intake of females (21 mg, t = -13.71, *p* = 0.000) and males (18 mg, t = -18.25, *p* = 0.000) was significantly below the RDA of 45 mg while only 6% of females and 02% of males met the RDA of Vitamin C. The thiamine intake of females (0.5 mg) was significantly below the RDA of 1.1 mg (t = -48.0, *p* = 0.000) while that of males (0.6 mg, t = -18.3, *p* = 0.000) was significantly below the recommended (1.2 mg), hence, none of females and males met with the requirements. Further, the Riboflavin intake of females (0.5 mg, t = -24.0, *p* = 0.000) was significantly below the RDA of 1.1 mg, and that of males (0.5 mg, (t = -33.5, *p* = 0.000) was significantly below the recommendations (1.6 mg). Further, only 2% of females met the RDA, while none of the males met the RDA of Riboflavin intake. The folate intake of females (180 μg) and males (198 μg) was fulfilled only 45% and 49.5% of RDA, respectively. Therefore, none of the female or male undergraduates met the RDA of folate intake. Similarly, none of the females and males met the RDA of Vitamin B6 intake and Vitamin B12 intake. The vitamin B6 intake of females (0.8 mg, t = -20.0, *p* = 0.000) and males (0.9 mg, t = -12.17, *p* = 0.000) was significantly below the RDA of 1.3 mg, while Vitamin B12 intake of females 1.4 μg (t = -16.0, *p* = 0.000) and males 1.3 μg (t = -16.7, *p* = 0.000) also significantly below the RDA of 2.4 μg. However, 73% of females and 66% of males met the RDA of Niacin intake with a mean intake of 16 mg and 18 mg, respectively (Table 3).

The total energy intake of those with BMIs underweight, normal, overweight, and obese was lower than the recommended dietary intake (2000 kcal for females, 2400 kcal for males). Further, the mean intake of protein was within the recommended value in the obese, overweight and normal-weight BMI population, while the underweight showed a low protein intake (43 g). The carbohydrate intake was higher in all BMI ranges than the recommended value, while the intake of obese > overweight > normal > underweight. However, the % E contribution through carbohydrates for all BMI categories ranged from the mean 60 – 58%. Added sugar was within the recommendation of all BMI categories while the mean saturated fat was higher than the recommendation ranging between 20 -21%. Further, the total fat was within the recommended values in all BMI categories and showed a low intake of dietary fibers as compared to the recommendations (Table 4).

**Table 4:** Macronutrient intake of sport undergraduates with underweight, normal, overweight, and Obese BMI

Macronutrient	Mean ± SD intake			Actual intake
	**Underweight** **(*n* = 37)**	**Normal** **(*n* = 55)**	**Overweight** **(*n* = 8)**	**Obese** **(*n* = 1)**
Energy, kcal/day	1552 ± 330	1681 ± 325	1800 ± 463	1789
Protein, g (RDA)	43 ± 9	46 ± 10	53 ± 15	54
Protein, % E (AMDR)	11 ± 1	11 ± 1	11 ± 2	10
Carbohydrate, g (RDA)	241 ± 57	268 ± 54	276 ± 64	279
Carbohydrate, % E (AMDR)	58 ± 4	60 ± 3	58 ± 6	58
Dietary Fiber, g (AI)	9 ± 6	9 ± 6	12 ± 8	8
Added Sugars, % E (DGR)	6 ± 4	5 ± 3	5 ± 3	8
Total Fat, % E (AMDR)	31 ± 4	29 ± 3	31 ± 5	31
Saturated fat, % E (DGR)	21 ± 3	20 ± 3	21 ± 5	21

The iron intake of all BMI groups was in the range of the iron intake of men, yet much below that is required by females (19.6 mg). The zinc level was high as compared to female intake while the normal weight group met with the recommendation for men. The intake of selenium and sodium was high in all BMI categories than the recommended while, iodine, Thiamin, Riboflavin, Folate, and vitamins A, B6, B12, C, D, and E were below the recommendation in all BMI groups. The intake of niacin is within the range of the underweight category, while that of normal and overweight was higher while that of the obese group is low (Table 5).

**Table 5:** Micronutrient intake of sport undergraduates with underweight, normal, overweight, and Obese BMI

Micronutrient	Mean ± SD intake			Actual intake
	**Underweight** **(*n* = 37)**	**Normal** **(*n* = 55)**	**Overweight** **(*n* = 8)**	**Obese** **(*n* = 1)**
Calcium, mg	411 ± 139	427 ± 130	376 ± 104	473
Magnesium, mg	175 ± 38	191 ± 47	174 ± 35	165
Potassium, mg	1440 ± 339	1533 ± 307	1515 ± 304	1610
Sodium, mg	2265 ± 551	2516 ± 541	2490 ± 751	2898
Iron, mg	9 ± 2	9 ± 2	9 ± 3	10
Zinc, mg	6 ± 1	7 ± 1	6 ± 1	6
Selenium,μg	43 ± 17	49 ± 15	44 ± 13	51
Iodine, ug	57 ± 17	59 ± 21	60 ± 27	60
Vitamin A, ug	402 ± 431	332 ± 140	425 ± 139	284
Thiamin, mg	0.5 ± 0.2	0.5 ± 0.1	0.5 ± 0.1	0.6
Riboflavin, mg	0.5 ± 0.2	0.5 ± 0.2	0.4 ± 0.2	0.5
Niacin, mg	15 ± 3	17 ± 4	18 ± 4	12
Folate, μg	180 ± 51	188 ± 54	221 ± 43	103
Vitamin B6, mg	0.8 ± 0.1	0.8 ± 0.2	0.9 ± 0.2	0.7
Vitamin B12, μg	1.3 ± 0.5	1.4 ± 0.4	1.5 ± 0.5	1.4
Vitamin C, mg	17 ± 8.3	21 ± 14	23 ± 14	8.5
Vitamin D, μg	0.8 ± 0.5	0.9 ± 0.8	1.3 ± 1.2	0.7
Vitamin E, mg	1.6 ± 0.6	1.8 ± 0.8	2.2 ± 1.2	1.9

## Discussion

Nutrition plays an important role in maintaining growth, good health, and providing energy. Sports nutrition is essential to maintain and increase athletic performance and reduce the risk of injury and increase the recovery rate [29, 30]. Our present study is an initiative to report the nutritional status of the undergraduates studying sport science and physical education degree programs and who are being trained to take the leadership in the field of sports. According to American Dietetic Association, and Hassapidou, & Manstrantoni 2001, athletes must consume adequate nutrients to provide energy 1800—2000 kcal/day [31, 32], and according to DGA it is 2000 kcal/day for females, 2400 kcal/day for males to maintain optimum body composition and BMI. The undergraduates following the Sport degree programs are involved in compulsory physical activities at least for three hours per day, Monday through Friday [33]; hence the daily energy intake must be sufficient to fulfil the energy requirement for a moderately intense level of training throughout the program, which is greater compared to a normal healthy individual. Previous studies performed on a general adult population aged between 18 to 30 years in Sri Lanka, the energy intake was reported to range 1942—2392 kcal/day in males and 1108 – 2052 kcal/day in females. However, the energy intake of female sports undergraduates (1607 ± 365 kcal/day) was also relatively low comparatively to that was reported previously [34]. Further, when compared to the lowest recommended energy intake for athletes 1800 kcal [31, 32] the female undergraduates on average showed an energy deficit of 193 kcal/day while the male undergraduates showed a deficit of 77 kcal/day.

The importance of proper dietary intake of both macronutrients and micronutrients in maintaining good health has been emphasized by many researchers [35–37]. Our study population exhibited that their intake comprises mostly carbohydrates, high in saturated fats, and less intake in protein and dietary fibers agreeing with the previous findings performed on the nutritional status of undergraduates [34, 38, 39]. Furthermore, supporting the previous finding, this study also revealed that the active females have less macro and micro-nutrients; hence was insufficient to fuel their training [31, 40].

Carbohydrates are the most preferred macronutrient stored as glycogen in muscles and used to fuel muscular activity. Further, the recovery from exhaustion due to exercise is also facilitated by a rich carbohydrate diet [41]. Both males (273 ± 49 g) and females (252 ± 61 g) showed a significant increase in carbohydrate intake in comparison to the DGA’s RDA recommendations (130 g). However, according to the recommendation of ACSM as Dietary Guidelines for Athletes, 6—10 g of Carbohydrates / Kg. Bw/ day was not achieved by most of the females (81%) and males (89%) who participated in this study. Further, a diet with energy less than 2000 kcal/day, a diet with 60% of carbohydrate intake is not sufficient for the optimal carbohydrate store of the athletes to maintain liver and muscle glycogen stores in their body [31, 40]. Further, this study revealed that both males and females reached the nutritional goals recommended for the % E by carbohydrates as prescribed by DGA’s AMDR and WHO.

Protein intake is essentially required for building muscle mass, and the amino acids from dietary protein are required for the biosynthesis of hormones and enzymes and to build the immune system [42–44]. Our studies showed the sport undergraduates did not meet the recommended allowances for their protein intake according to DGA’s RDA, yet were within the range for the recommendations of % E proteins provided by DGA’s AMDR and WHO for a general population noting to be closer to the lower end. However, when comparing with the recommendation for athletes 1.2 – 1.7 g/kg BW/day [45], the athletes with a 50 kg body weight and involved in moderate intense or high-intensity training must consume proteins in the range of 60 to 85 g/day. According to the finding through our studies majority of both females (92%) and males (95%) did not meet the recommended intake of proteins required for moderately intense athletes revealing that their protein intake is not adequate to meet their physical activity levels.

Fats are also an important macronutrient in maintaining good health and act as a metabolic fuel during enedurance exercise and performance [46]. Our results showed that all undergraduates' fat intake was at an acceptable range for athletes and a considerable proportion of both females (55%) and males (39%) undergraduates reported a higher fat intake than the recommendation for the general population according to the DGA’s ADMR and WHO recommendations indicating that the food preparation methods, type of food consumes must be monitored more closely.

The overconsumption of saturated fats has reported to decrease the athletic performance and poor recovery [47]. Therefore, the consumption of saturated fats should not exceed 10% of the daily caloric intake [25, 26]. The saturated fat intake of all undergraduates was higher than the recommendations and could be due to the consumption of coconut oil and coconut milk as they are the main ingredients in Sri Lankan dishes [48, 49].

A sufficient dietary fiber intake is reported to reduce the risk of non-communicable diseases [50]. Our Studies also support the findings of previous studies on the Sri Lankan adult population that reported higher saturated fat intake and low dietary fiber intake [34, 39].

Micronutrients (vitamins and minerals) play a crucial role in maintaining metabolism and tissue functions, and therefore deficiencies in daily dietary intake could lead to many diseases. Our study showed that the micronutrient intakes in vitamin C, B1, B2, B9, B6, and B12 and minerals such as Ca, Mg, and K were low in both male and female sport undergraduates while the females on average exhibited a low intake in iron. Of the B vitamins, B1, B2, B3, B6, B9, and B12, both male and female undergraduates met the requirement only in Vitamin B3 (niacin). Niacin is predominantly present in brown rice and to a lesser extent in polished rice [51, 52]. In general, the Sri Lankan population consumes a large amount of rice in their meals and most people consume rice for all three meals in the day [53, 54]. It is most likely that the increased consumption of rice supports sufficient niacin intake in their diet. However, results indicate our study population’s dietary intake is deficient in B vitamins which are important in carbohydrate and protein metabolism. Therefore, it is possible that above deficiencies could lead to impairments in producing energy to fuel and recover from physical activities [55, 56].

Furthermore, the above information indicates that the undergraduates' accessibility to vegetables and fruits is substantially low, similar to that was reported previously [57]. Hence, measures must be implemented to ensure the students have a sufficient nutritionally balanced diet through the cafeterias. However, only nine percent (09%) of females reported consuming the daily required vitamin A (500 μg) while only 05% of males met the recommended intake of 600 μg. Our investigations revealed that none of the undergraduates met the dietary requirement in both Ca and Vitamin D, which are essential nutrients for sports undergraduates to maintain their bone health.

Our study revealed that only 02% (1 female) reported an iodine intake of more than 150 μg, while none of the males achieved the recommendation. The deficiencies in iodine leading to dysfunctional thyroid glands will result in fatigue, low stamina, lethargy, muscle aches & cramps, muscle weakness, joint pains, and poor memory & brain functions, leading to many difficulties in regular training of athletes.

This study provided us with details of both macro and micronutrient deficiencies among sport science undergraduates. Therefore, firstly the students must be made aware of the importance of a nutritionally balanced diet and its impact on maintaining and improving their academic, mental, and physical performances. Further, these results can be used in the formulation of policies and in designing special diets to suit the undergraduates reading for the sport science degree program. Hence, the undergraduates must be encouraged to have a diet comprising fruits, milk, meat, and vegetables regularly with a low salt diet and less saturated fats. The above deficiencies and excessive intakes of nutrients such as carbohydrates, sodium, saturated fats etc. emphasize the fact that there must be direct interventions and proper dietary guidelines implemented specially among the undergraduates of the sport science degree program, ensuring they receive proper and guided nutrition to maintain their good health.

However, our results indicate despite daily course-related physical activities daily, 11% of males and 06% of females were overweight, with only 02% of females (01) obese. Further, 35% of male and 38% of female undergraduates were below the expected range of BMI (underweight). Therefore, collectively our findings reveal although all undergraduates in this study met the dietary requirements of carbohydrates and consumed fats above the recommendations there is a considerable number of undergraduates underweight.

In general, BMI which is below normal is generally associated with low intakes of total energy intake, proteins, carbohydrates, dietary fiber, and fats [58]. Obesity is associated with a high intake of simple carbohydrates and a low intake of complex carbohydrates [59, 60]. However, in our study, all participants at all BMI levels showed a low energy intake, higher carbohydrate, high fat, low protein, and low dietary fiber intakes as compared to the Dietary Guidelines for Americans. Supporting the observations of, Martinchik et al., 2020 our study too showed that the obese and the overweight groups in men showed carbohydrate-associated lower energy consumption [61].

High intake of calcium, magnesium, potassium, zinc and iron has been shown to reduce BMI supporting the weight loss of overweight and obese individuals [58, 62–65]. Further, a high intake of sodium is associated with weight gain irrespective of the total calorie intake [66]. Our study too revealed although the total energy intake was low in both obese and overweight groups their sodium intake was high [66]. This could be an indication of those who are overweight and obese consume fast-processed food to meet their daily dietary intake[67].

## Strengths and limitations

Of all the undergraduates following Sport Sciences, Physical Education, or sport related degree programs around 67% of the sports undergraduates are registered at the Sabaragamuwa University of Sri Lanka. Our study population does not represent the sports undergraduates who are studying at other universities in Sri Lanka yet represent the majority in the sport-related degree programs. Universities offering sport-related degree programs have different accessibility to food by type, quantity, and diversity. Hence the majority of the Sri Lankan sport undergraduate population (67%) studying at SUSL, have uniformity in accessibility to food, same environmental condition, and uniform curriculum and academic and physical activities minimizing the variabilities that influence the results of this study. As with any other dietary intake assessment method, the three-day food-dairy method has its limitations in measuring the dietary intake during two weekdays and one weekend. However, this method imposes less burden on the responders compared to the 7-day food-dairy method resulting in a higher participant completion hence recommended for a population with a busy daily schedule. It is important to note that there is potential to underreport or overreport. Such responses were excluded from the study as outliers.

## Conclusion

The study revealed that the undergraduates' dietary intake has deficiencies in macronutrients such as proteins and micronutrients such as vitamin C, B1, B2, B9, and B12 and minerals such as Ca, Mg, and K, which could negatively influence their academic and physical performance. Therefore, proper dietary recommendations must be made along with improving the accessibility to a nutritionally balanced diet for undergraduates. Therefore, to maintain the nutritional status of sport undergraduates, we recommend to the Vice-chancellor and the Dean to establish special cafeterias for sport undergraduates to provide nutritionally balanced meals, perform regular food quality audits, and perform clinical check-ups to ensure the students have maintained proper nutritional status thereby maintaining proper physical and mental health.

## Data Availability

All data sets, information and the statistical analysis that has been used to compile the findings stated in this manuscript is available and can to obtained from the corresponding author, Prof. M. Nirmali Wickramaratne upon request.

## Abbreviations

AMDR: Acceptable Macronutrient Distribution Range
AI: Adequate Intake
DGA: Dietary Guidelines for Americans
DGR: Dietary guidelines recommended limit
RDA: Recommended Dietary Allowance
UL: Upper Limit
WHO: World Health Organization
